# Iran’s Health Reform Plan: Measuring Changes in Equity Indices

**Published:** 2018-03

**Authors:** Abbas ASSARI ARANI, Tohid ATASHBAR, Joseph ANTOUN, Thomas BOSSERT

**Affiliations:** 1. Dept. of Development and Planning, Faculty of Management and Economics, Tarbriat Modarres University, Tehran, Iran; 2. Dept. of Health Economics, Faculty of Management and Economics, Tarbriat Modarres University, Tehran, Iran; 3. Center for Health Policy, Harris School of Public Policy, University of Chicago, Chicago, USA; 4. Dept. of Global Health and Population, Harvard T.H. Chan School of Public Health, Harvard University, Boston, USA

**Keywords:** Health policy, Policy evaluation, Health equity, FFC, CHE, IHE, Health sector reform

## Abstract

**Background:**

Two years after the implementation of the Health Sector Evolution Plan (HSEP), this study evaluated the effects of the plan on health equity indices.

**Methods:**

The main indices assessed by the study were the Out-of-Pocket (OOP) health expenditures, the Fairness in Financial Contribution (FFC) to the health system index, the index of households’ Catastrophic Health Expenditure (CHE) and the headcount ratio of Impoverishing Health Expenditure (IHE).

**Results:**

The per capita share of costs for total health services has been decreased. The lowered costs have been more felt in rural areas, generally due to sharp decrease in inpatient costs. Per capita pay for outpatient services is almost constant or has slightly increased. The reform plan has managed to improve households’ Catastrophic Health Expenditure (CHE) index from an average of 2.9% before the implementation of the plan to 2.3% after the plan. The Fairness in Financial Contribution (FFC) to the health system index has worsened from 0.79 to 0.76, and the headcount ratio of Impoverishing Health Expenditure (IHE) index deteriorated after the implementation of plan from 0.34 to 0.50.

**Conclusion:**

Considerable improvement, in decreasing the burden of catastrophic hospital costs in low income strata which is about 26% relative to the time before the implementation of the plan can be regarded as the main achievement of the plan, whereas the worsening in the headcount ratio of IHE and FFC are the equity bottlenecks of the plan.

## Introduction

To improve the health system, Iran’s government has been carrying out the Health Sector Evolution Plan (HSEP) or Health Reform Plan (HRP) since 2014. Various purposes have been mentioned in this plan, the most important of related to healthcare costs as follows:

- Reduction of direct payment of out-of-pocket expenses for people receiving healthcare services.
- Reduction of the percentage of households who incur heavy expenses for receiving health services.
- Establishment of equality in payments for utilization of healthcare services.

According to various official documents of the plan (1,2), HSEP has been implemented in eight separate programs as follows:

- Reduction of the share paid by patients hospitalized in public hospitals
- Support for the stay of physicians in underserved areas
- Presence of resident physicians in public hospitals
- Promotion of hospitality quality in public hospitals
- Improvement of the quality of visitation services in public hospitals
- Promotion of natural childbirth
- Financial protection of poor patients with incurable or special diseases
- Supervision of the good implementation of health reform programs

According to the official documentation of the plan, the eight programs of HSEP have been implemented in three steps:

- Extending insurance coverage, decreasing insurer out-of-pocket expenditure, and improving the quality of hospital services,
- Prevention of patients from referring outside hospitals to receive equipment and laboratory services,
- Updating the book of tariffs on medical services.

The present study evaluated the extent to HSEP achieved its objectives in this area by assessing the main indicators related including out-of-pocket (OOP) payments, catastrophic health expenditure (CHE), impoverishing health expenditure (IHE) and fairness in financial contribution (FFC) to health system index, in various income deciles of urban and rural areas before and after the implementation of the plan.

## Methods

The most comprehensive and oldest microdata in Iran’s statistical system titled the household income-expenditure plan has been annually developed and published by the Statistical Center of Iran for 50 years. Improved based on the United Nations statistical recommendations (3) and the international system of national accounts (SNA) (4, 5), this plan is conducted using surveys by visiting sample households in urban and rural areas. The statistical unit in this plan is a typical Iranian household living in an urban or rural area. Statistical times for different questions include previous week, previous month, and the last 12 months. The survey is started during May through the end of March of the next year.

The sampling volume in the survey is sufficient to reach 95% confidence level in calculations about Out-of-Pocket health expenditures and the related indices (including catastrophic health expenditures, headcount ratio of health impoverishing expenditures and financial fairness in health expenditures).

The amount of out-of-pocket expenses paid by households for receiving healthcare services was extracted and presented for different deciles and rural or urban areas. The study conducted from Dec 2015 to mid-Mar 2017. For the ease of comparison, the data related to two years before the plan and were compared to the data of two years after the plan. Then, the catastrophic health expenditure, as defined by the WHO, was extracted and presented. Based on this definition, spending more than 40% of the household payment, capacity on medical expenses is evaluated as an unfavorable status, and such families are so-called households at risk of unaffordable or catastrophic costs (Equation 1).

Equation 1 $$\begin{array}{c} {\mathrm{oopctp}}_{h}\geq 0.4\;\;\;\mathrm{if}\;\;\;{\mathrm{cata}}_{h}=1\\ {\mathrm{oopctp}}_{h}<0.4\;\;\;\mathrm{if}\;\;\;{\mathrm{cata}}_{h}=0 \end{array}$$

In terms of measuring fairness in the financial burden of health costs on individuals, a theoretical framework provided by WHO is based on the approach that a health system is fairly financed when all households dedicate the same share of their capacity or ability to pay (effective non-subsistence income) to receiving the required healthcare services. This index was formally introduced by WHO in its annual report of 2000 (6, 7) and was used and developed subsequently by many researchers (8–16). According to this approach, WHO defined the index of the FFC, formulated as follows:

Equation 1:

$$\mathrm{FFC}=1-\sqrt{\frac{\sum_{h=1}^{n}w_{h}/{\mathrm{oopctp}}_{h}-{\mathrm{oopctp}}_{0}{/}^{3}}{\sum w_{h}}}$$

In this equation, FFC, *oopctp*_0_, and *oopctp*_*h*_ denote the Fair Financial Contribution Index, the sum of out-of-pocket payment for healthcare services (OOP), divided by the sum of capacity to pay, and the ratio of out-of-pocket payment to capacity to pay, respectively. However, some items, such as the cost of meals in the hospital or patient transfer to the hospital, are excluded from the medical expenses paid by households. Ultimately, *w_h_* represents the weight added to the equation for analysis at the national level to modify the sample according to the population features.

Depending on the definition of cost, the FFC can be calculated in three ways, as follows:

- by merely calculating the household consumption expenditures (the most common and interpretable definition used for the FFC);
- by calculating the household consumption and non-consumption expenditures (durable goods); and
- by calculating the household consumption and non-consumption expenditures and investment costs.

Because of the possible difficulties of interpretation and calculation, gross values are used in the calculation of durable goods and investment costs.

In addition to FFC and CHE, the headcount ratio of impoverishing health expenditure (IHE) also is regarded as one of the main indicators related to poverty and equity in healthcare services. This index indicates how many families fall under the poverty threshold due to healthcare expenditure. The poverty line can be calculated in different methodologies, the two most common methods are “dollar-a-day (DAD) poverty line” (Based on food, clothing and accommodation expenditure data, produced and used in World Bank reports) and the “subsistence level food expenditure poverty line” (Based on food expenditure data, used in some WHO reports).

The idea behind DAD is to estimate poverty line in terms of absolute poverty in the world’s poorest regions also matched to the same real level of welfare in other regions. The latter requirement has led to use purchasing power parity exchange rates (PPPs) to convert the poverty line into the US dollar and, into the currency of each country, during the estimation of calculation of global poverty line (17). World Bank’s international poverty line (IPL) index uses updated data about food, clothing, and shelter needs to reflect a more accurate picture of the costs of living. The newest IPL is $1.90 (the previous line was $1.25). In other words, the real value of $1.90 in today’s prices is the same as $1.25 was in 2005.

The second method used in proposed in some WHO reports uses a more complicated methodology; “The poverty line is defined as subsistence level food expenditure estimated as the average food expenditure per equivalent adults of households in the 45th–55th food budget share distribution. When actual food spending falls below this amount, then capacity-to-pay is defined as total expenditures net of actual food spending” (18, 19).

The application of WB’s IPL is more common and prevalent in the health and equity literature, mainly because of its simple method of calculation, more comprehensible concept and globally comparable index.

We use the WB’s IPL; the absolute poverty threshold is equal to a daily income of 1.9 dollars (PPP), or an income of 57 dollars per month per person. In simpler words, if per person expenditure, including health care services exceed the poverty line of 57 dollars in a month (228 PPP dollars in a month for a four-person family), while the expenditure without healthcare services drop below the poverty threshold, the person or family faces impoverishing health expenditure.

## Results

**Fig. 1:**
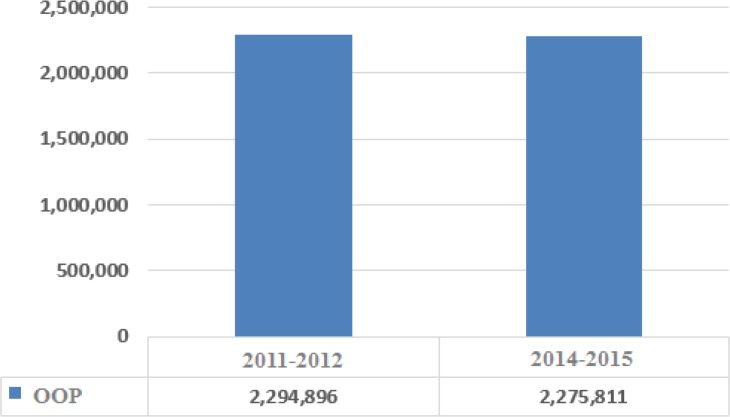
Per capita out of pocket health costs in real terms of 2011 before and after the implementation of the HSEP for all deciles (domestic currency)

**Fig. 2:**
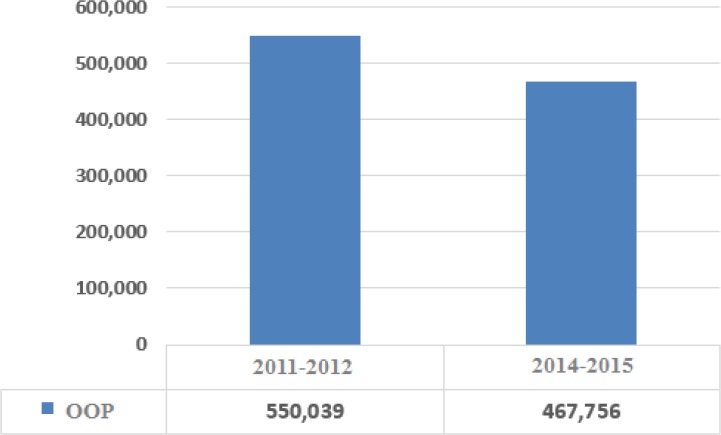
Per capita out of pocket health costs in real terms of 2011 before and after the implementation of the HSEP for decile 1 (domestic currency)

The research computations showed the per capita share of costs for total health services (in real terms of 2001) has been decreased for the entire population after the implementation of the plan (Fig. 1). The index has seen considerable decrease for the lower parts of the social stratum and slight increase in upper deciels (Fig. 2). The lowered costs have been more felt in rural areas, generally due to sharp decrease in inpatient costs. The research also finds that the per capita pay for outpatient services is almost constant or has slightly decreased.

On the other hand, calculation of catastrophic health expenditure indicates that the number of households under catastrophic health costs has reduced by 0.57% (from 2.92% to 2.35%) after the implementation of the HSPE, making for an improvement rate of 19.5%. Improvement of mean in lower deciles has been greater than that of the average deciles, as the mean of lower deciles has improved by about 26% (up to 0.76% absolute reduction). Improvement of catastrophic expenditures in urban areas has been slightly greater than rural areas. According to the findings, catastrophic health expenditure in urban and rural areas has shown a reduction of about 0.7% (21% improvement) and slightly more than 0.5% (about 18% improvement). The findings also indicate that Headcount ratio of Impoverishing Health Expenditure (IHE) index has worsened after the implementation of the plan from 0.34% to 0.5%.

The results of FFC calculations, using the household income-expenditure survey data before and after the implementation of the HSEP in rural and urban areas, have been shown in Table 1. Gray cells represent the duration of HSEP implementation and the cells in the second row show the FFC values in the most common and interpretable definition used for estimating FFC (including the household consumption expenditures).

**Table 1:** FFC values in the total population

***Definition of costs used in calculation of FFC/Year***	***2011***	***2012***	***2013***	***2014***	***2015***
Household costs	0.809	0.831	0.788	0.757	0.763
Household costs plus gross costs of durables, etc.	0.851	0.856	0.834	0.831	0.830
Household costs plus gross costs of durables, etc., plus investment costs	0.861	0.858	0.831	0.831	0.839

In the most common definition for calculating the FFC, the value of this index in rural areas, urban areas, and the whole population has reduced (worsened) from 0.78 to 0.75, 0.81 to 0.78, and 0.79 to 0.76, respectively. Accordingly, during the two years’ of HSEP implementation, the FFC has worsened by 3.1%, 3.5%, and 4.2% in rural areas, urban areas, and the whole population, respectively.

## Discussion

The HSPE, with the motto of justice in the costs of healthcare services and improvement of accessibility of deprived strata, has managed to improve the status of lower-income strata in terms of both out-of-pocket payments and catastrophic health expenditure. This improvement has been more prominent in rural areas than urban areas. By separating costs into inpatient and outpatient, an improvement has occurred for all income deciles in the area of inpatient services, with a further increase in low-income and rural groups. By contrast, the status of outpatient services has remained constant or slightly increased.

In addition, a reduction was observed in catastrophic health expenditure after the implementation of the HSPE, which was more prominent in lower-income deciles and urban areas.

Improving CHE indicator shows that the HSEP has prevented households from experiencing “abrupt” poverty situations, usually resulting from surgery operations, by decreasing the cost of public hospitals’ inpatient services expenditure.

However, based on the fluctuations in headcount ratio of IHE, HSEP has intensified the health caused poverty - “slow” impoverishment if it can be said - resulting from healthcare services. The co-movement of FFC and IHE can be attributed to the fluctuations in the cost of outpatient services.

## Conclusion

The HSPE was designed with the goal of improving health indicators, establishing equity in the use of health services and reducing health costs. This research was conducted with the aim of assessing the extent to which the plan had achieved the equity-enhancing dimension of the goals set. The results showed that in addition to lowering burden of total health costs per capita, the most significant improvement in terms of equity indices has been about the reduction of catastrophic health costs.

## Ethical considerations

Ethical issues (Including plagiarism, informed consent, misconduct, data fabrication and/or falsification, double publication and/or submission, redundancy, etc.) have been completely observed by the authors.
